# Structural Optimization of a High-Performance Green Sandwich Made of Sisal Reinforced Epoxy Facings and Balsa Core

**DOI:** 10.3390/polym16233341

**Published:** 2024-11-28

**Authors:** Bernardo Zuccarello, Francesco Bongiorno, Carmelo Militello

**Affiliations:** Department of Engineering, University of Palermo, Viale Delle Scienze, 90128 Palermo, Italy; francesco.bongiorno01@unipa.it (F.B.); carmelo.militello01@unipa.it (C.M.)

**Keywords:** green materials, sandwiches, biocomposites, sisal fiber, mechanical properties

## Abstract

Within the range of composite laminates for structural applications, sandwich laminates are a special category intended for applications characterized by high flexural stresses. As it is well known from the technical literature, structural sandwich laminates have a simple configuration consisting of two skins of very strong material, to which the flexural strength is delegated, between which an inner layer (core) of light material with sufficient shear strength is interposed. As an example, a sandwich configuration widely used in civil, naval, and mechanical engineering is that obtained with fiberglass skins and a core of various materials, such as polyurethane foam or another lightweight material, depending on the application. Increasingly stringent regulations aimed at protecting the environment by reducing harmful emissions of carbon dioxide and carbon monoxide have directed recent research towards the development of new composites and new sandwiches characterized by low environmental impact. Among the various green composite solutions proposed in the literature, a very promising category is that of high-performance biocomposites, which use bio-based matrices reinforced by fiber reinforcements. This approach can also be used to develop green sandwiches for structural applications, consisting of biocomposite skins and cores made by low-environmental impact or renewable materials. In order to make a contribution to this field, a structural sandwich consisting of high-performance sisal–epoxy biocomposite skins and an innovative renewable core made of balsa wood laminates with appropriate lay-ups has been developed and then properly characterized in this work. Through a systematic theoretical–experimental analysis of three distinct core configurations, the unidirectional natural core, the cross-ply type, and the angle-ply type, it has been shown how the use of natural balsa gives rise to inefficient sandwiches, whereas performance optimization is fully achieved by considering the angle-ply core type [±45/90]. Finally, the subsequent comparison with literature data of similar sandwiches has shown how the optimal configuration proposed can be advantageously used to replace synthetic glass–resin sandwiches widely used in various industrial sectors (mechanical engineering, shipbuilding, etc.) and in civil engineering.

## 1. Introduction

Thanks to their particular properties, composite sandwich laminates have found widespread use in numerous industrial sectors in recent decades. Originally developed in the aeronautical sector for their high strength and specific flexural stiffness characteristics, these materials are now also of considerable interest in the automotive, shipbuilding, transport, construction, and sports sectors [1,2]. They are released by connecting two thin skins made of a stiff and strong material through an interposed, softer, and less strong core.

In recent years, the growing sensitivity to environmental issues and the existence of specific regulations against environmental pollution have led to considerable interest among researchers in the so-called green composites that can be variously recycled at the end of their lives. These materials are generally composed of “bio-based” matrices having low environmental impact, reinforced by natural fibers. Among these, green sandwiches are of particular interest, as they are characterized by high flexural strength combined with low specific weight [3] and low environmental impact. They generally consist of strong biocomposite skins, which demand the flexural strength, and a core of very light and relatively low-strength green material (properly selected to withstand only the shear stresses associated with flexural).

The examination of the technical literature shows the presence of various classical studies [1,2] about sandwich panels made of conventional materials such as metal skins and foam cores, with particular interest in increasing the absolute and specific mechanical properties, but also the presence of more recent studies characterized by a growing interest in reducing the environmental impact of materials and structures through the search for environmentally friendly composites [4].

In [5], Hoto et al. investigated the flexural behavior and water absorption of a new asymmetric sandwich with a cork core and skins made of natural fiber-reinforced green composites, such as basalt and flax fiber. Their results have shown very good behavior in terms of energy absorption during flexural tests, whereas the water absorption of the samples was significantly reduced by the infiltration of resin into the core. These performances confirm how such a green sandwich can be a good alternative to traditional ones.

Also in [6], flax and basalt fibers have been used for the reinforcement of green hybrid composite sandwich structures based on cork. The results of such research work have shown that the position of the basalt fibers plays a key role in the flexural failure of the sandwich structures due to the differences in stiffness between flax and basalt fibers.

In [7], flax and jute fabrics were used as reinforcements with polyester resin to form eco-friendly composite skins, whereas poplar particleboard was used as the core to make composite sandwich structures using the vacuum-assisted resin transfer molding technique. The obtained results have shown that the engineered panels proposed by Mohareb et al. have high mechanical properties, suitable for various structural applications.

Other researchers have also employed flax fiber to make green composites for sandwich structures [8,9].

In [10], the authors consider skins that consist of epoxy matrix composites (based on tree sap) reinforced with woven hemp fabric and a core consisting of castor oil-based polyurethane foam reinforced with rice husk ash. The results obtained by numerical simulations are in good agreement with experimental tests; they have shown that such biocomposite sandwiches can replace conventional plasterboard panels.

Instead, a new green bioepoxy composite reinforced with basalt fibers (BFR) is proposed in [11] as an environmentally friendly alternative to traditional petroleum-derived composites. This biocomposite was combined with cork as a base material for the fabrication of sandwich structures used to produce a ‘longboard’ as a case study of a sports equipment application.

In addition, with regard to the core of sandwich structures, some researchers propose the use of innovative 3D printing methods to obtain structural cores made of PLA [12] or cellular panels made of wood fiber/PLA biocomposite [13].

However, the increased use of environmentally sustainable materials has led to new opportunities for balsa to replace non-renewable petroleum-based cores, such as polymer foams used in a wide range of sandwich structures [14].

In order to propose a green, high-performance composite sandwich that can be used to replace synthetical composite materials, a new configuration consisting of high-performance sisal–epoxy biocomposite skins and an innovative renewable core of balsa wood laminates with appropriate lay-up has been developed and accurately characterized in this work. The use of a material characterized by extreme lightness and total renewability for the core, such as common balsa wood, has been proposed in an original innovative laminar configuration that significantly improves the performance of the sandwich, permitting also to avoid the critical premature damage phenomena, such as indentation, skin buckling, and skin-core delamination, that occur when using natural unidirectional balsa. In such a manner, the optimal sandwich configuration proposed is characterized by mechanical performance comparable with the best sandwich proposed in literature, as confirmed by accurate comparisons.

## 2. Materials and Methods

### 2.1. Materials

As briefly said above, the primary objective of the present work is to exploit the high-performance biocomposite laminates developed by the same authors in [15,16,17,18,19,20,21,22,23,24] to manufacture proper skins that allow obtaining an innovative high-performance green sandwich. In detail, these are high-quality green laminates obtained by an optimized compression molding process [17] and consisting of a green epoxy matrix reinforced by long sisal fibers. Balsa wood has been properly selected for the core to exploit its extreme lightness and total renewability.

In detail, the following Table 1 shows the main characteristics of the materials selected for the green sandwich to be implemented:

(1) sisal fibers are supplied by Mellau-Teppich Lotteraner, Wüstner GmbH & Co KG (Mellau, Austria) with a low specific weight of 1.45 g/cm^3^, tensile strength of 685 MPa, Young’s modulus of about 40 GPa, and ultimate tensile strain of 1.75%;
(2) balsa wood with a specific weight of 150 g/cm^3^ (the other characteristics are not provided by the vendor and will be determined in the following);
(3) green epoxy resin called SUPERSAP CLR with SuperSap INH Hardener (San Antonio, CA, USA), which, as amply demonstrated in previous studies by the same authors, has an almost linear elastic behavior with a specific weight of 1.05 g/cm^3^, tensile strength *σ_m_*_,*R*_ of about 50 MPa, Young’s modulus *E_m_* equal to 2.5 GPa, and an ultimate tensile strain *ε_m_*_,*R*_ of 2.5%.

### 2.2. Cores Manufacture

Along with lightness and renewability, balsa has an almost uniaxial fibrous structure with low transverse shear and compressive strength, so it is routinely used in the manufacturing of medium/low strength sandwiches for light marine applications, commonly consisting of balsa cores and skins of conventional wood laminates. In its natural configuration, therefore, balsa is not very strong for the implementation of a high-performance green sandwich, such as the one to be implemented in the present work. The typical presence of fibers parallel to the sandwich axis, in fact, is quite unuseful in terms of shear strength and transversal compressive strength of the core. In order to retain the lightness and renewability of balsa but to overcome both these strength limitations, an innovative home-made laminar configuration is properly proposed for the core.

In more detail, in order to maximize both the shear strength and the transversal compressive strength, the laminate lay-up [(±45/90)*_n_*] is proposed; as it is well known from the micromechanics, in fact, the laminae arranged at ±45° permit increasing the shear strength, whereas the laminae arranged at 90° permit increasing the transversal compressive strength, since in such a manner the balsa fibers are always oriented in the directions of the maximum/minimum stress due to shear load and transversal load, respectively.

**Figure 1:**
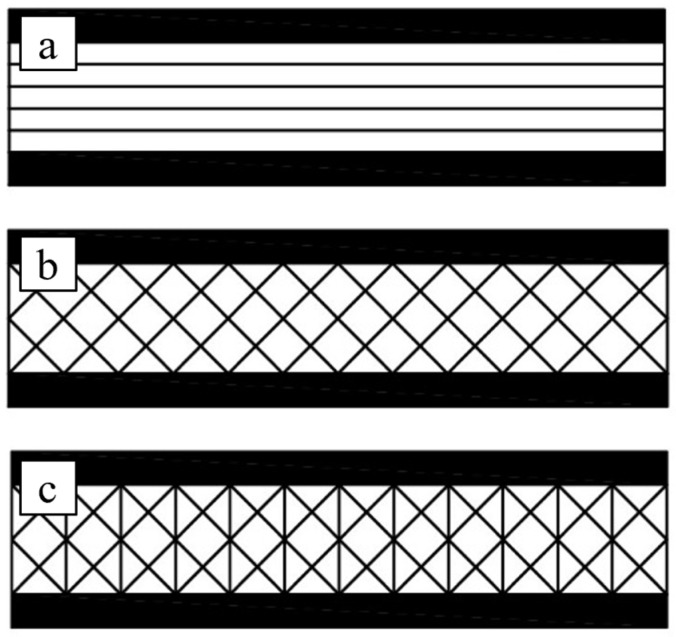
Balsa core with fibers at (**a**) 0°—called B(0), (**b**) ±45°—called B(±45), and (**c**) ±45°/90°—called B(±45/90).

In order to verify the actual improvements achieved by the innovative laminar configuration proposed for the balsa core compared to the natural unidirectional single layer configuration (see Figure 1a) and also to verify if a simpler cross-ply configuration such as [(±45)*_n_*] is sufficient to achieve the required shear and transverse compression performance, all three configurations defined as B(0)—Figure 1a, B(±45)—Figure 1b, and B(±45/90)—Figure 1c are considered in the present work.

**Figure 2:**
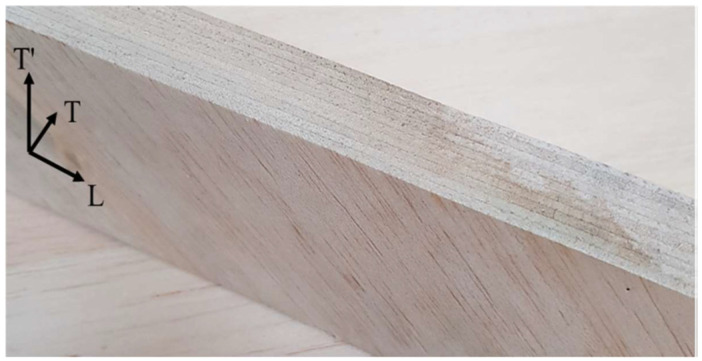
Innovative balsa laminate core type B(±45/90).

The two laminar configurations, B(±45) and B(±45/90), have been obtained by laminating thin balsa sheets, about 2 mm thick, with a vertical lamination plane. The sheets were carefully bonded together with the same green epoxy resin used for the skins as described above. The proposed laminated core B(±45/90), so obtained, is shown in the following Figure 2.

### 2.3. Skins Manufacture

High-performance unidirectional laminate skins with a volume fraction *V_f_* = 55% have been manufactured by using the optimized compression-molding process already described in detail in previous work [17], to which the reader is referred for further details. This manufacturing process allows high-quality laminates (low concentration of voids and defects) to be obtained by using a 100-ton hydraulic press. Using unidirectional 200 g/m^2^ stitched fabrics specially produced in the laboratory, unidirectional panels of approximately 4 mm thickness were obtained with lay-up [0_16_].

### 2.4. Sandwichies Manufacture

**Figure 3:**
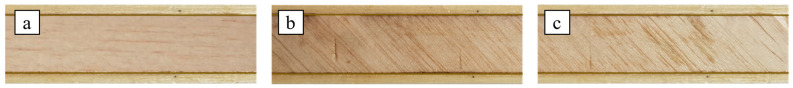
Sandwich samples manufactured in the laboratory: (**a**) SB(0), (**b**) SB(±45), (**c**) SB(±45/90).

Using the skins and the three core types [B(0), B(±45), and B(±45/90)] described in the previous chapters, three types of sandwiches have been obtained by simply gluing the skins and core together, still using the green epoxy resin used as the matrix of the skins. In the first type of sandwich, called SB(0), the core consists of a simple balsa panel with longitudinal fibers (see Figure 3a); in the second type, called SB(±45), the core consists of a cross-ply balsa laminate with lay-up [±45°] (see Figure 3b); in the third type, called SB(±45/90), the core consists of an angle-ply balsa laminate with lay-up [±45/90] (Figure 3c).

## 3. Experimental Results

The preliminary characterization of skins and cores and the subsequent analysis of the mechanical behavior of the three types of sandwiches considered were performed by using a 30 kN MTS 793 servo-hydraulic test machine.

### 3.1. Tensile Characterization of Skins

**Figure 4:**
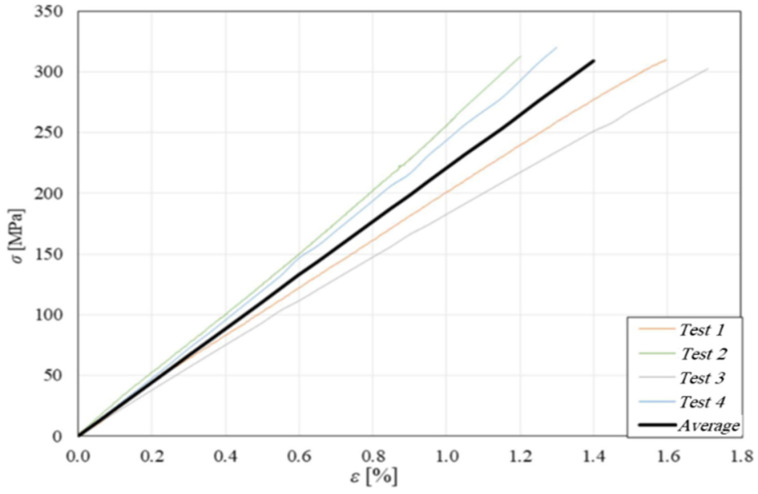
Longitudinal tensile curves for four skin specimens tested and their average curve.

The mechanical behavior of the skins has been determined through tensile tests performed in accordance with ASTM standard D3039 [25], monitoring the strains with a knife extensometer having a 25 mm measuring base. The following Figure 4 shows the result of the tensile tests performed on 4 separate specimens, together with the corresponding average curve. The observation of these curves shows a linear elastic behavior of the skins until fracture, which occurs with an ultimate tensile stress $\sigma_{s,u}\;$ = 310 MPa, a failure strain of about 1.4%, and a longitudinal modulus of *E_s_* = 22 GPa.

As extensively shown in previous works [15,16,17,18,19,20,21,22,23,24], the unidirectional biocomposite considered is characterized by a compressive strength comparable to the tensile strength determined here in detail for the material actually used, so no further compressive tests have been performed, and $\sigma_{s,u}$ = $\sigma_{s,u}^{’}$ has been considered.

### 3.2. Transverse Compression Characterization of the Core

**Figure 5:**
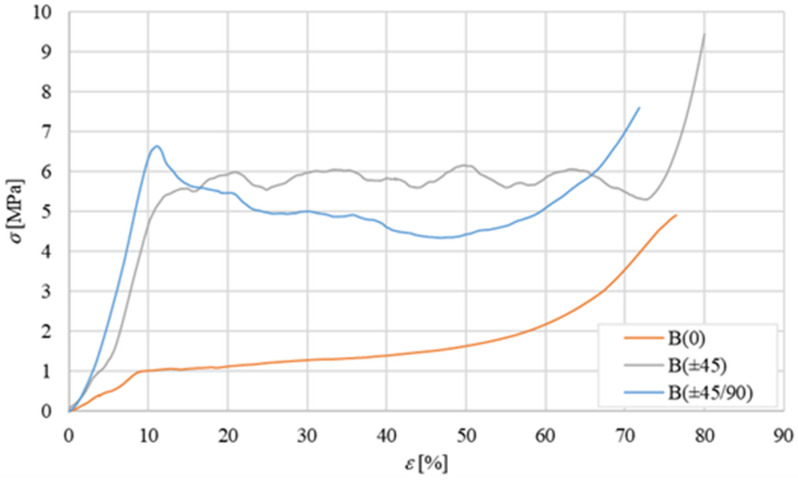
Average flatwise compression curves of the tested specimens B(0), B(±45), and B(±45/90).

Considering that, due to the relatively high shear stiffness of the skin with respect to the core, the shear characterization of the core results in practice in the simple shear characterization of the sandwich, the characterization of the simple core has been carried out by considering only the so-called flatwise (‘transversal’) compression test, leaving the shear characterization of the core to the following shear characterization of the sandwich according to the ASTM C365-57 [26]. The average flatwise compression curves obtained for the three core configurations B(0), B(±45), and B(±45/90) are shown in Figure 5 below:

The curve for the B(0) core shows a first linear segment between 0 and 1 MPa, followed by a second part having an almost zero slope (plateau at about 1 MPa until a strain of about 20%), followed again by a third part having a progressively increasing slope until fracture. As it is known, the first linear part corresponds to the elastic compressive deformation of the material, the second part represents the phase of compressive instability of the intimate structure of the material (micro-buckling), and the third part corresponds to the progressive resumption of the post-buckling load due to densification phenomena of the material. The stress reached at the end of the elastic section, to be considered as the failure stress of the material, is equal to approximately $\sigma_{c,R}\;$ = 1 MPa. This corresponds to a percentage of elongation $\varepsilon_{c,R}$ ≅ 9%. The compressive stiffness of the balsa corresponding to the elastic field (first part of the curve) is therefore equal to *E* ≅ 10 MPa. A similar curve is exhibited by the laminar core type B(±45); the stress reached at the end of the elastic section, which is always to be considered as the compressive failure stress, is now equal to about $\sigma_{c,R}\;$ = 5.6 MPa. The compressive stiffness of this core obtained in the elastic field (from the first part of the curve) is instead equal to *E* ≅ 39 MPa. Also, core type B(±45°/90°) exhibits a similar three-part curve in which the initial linear trend continues until a stress peak equal to $\sigma_{c,R}\;$ = 6.6 MPa. The compression stiffness of such a core in the elastic field (first part of the curve) is equal to *E* ≅ 59 MPa. Such results are qualitatively in good accordance with the well-known Classical Theory of Laminates (CTL, see ref. [27]); they are essentially due to the progressive alignment of the fibers with the applied compressive load, which occurs by passing from the simple balsa with unidirectional fibers lying all at 0° to the presence of fibers at ±45° (fibers partially aligned with the applied load) and also at 90° (fibers perfectly aligned with the applied load), that contribute to improving both the stiffness and the compressive strength of the core.

In summary, therefore, it can be observed how the transition from the natural unidirectional B(0) configuration of the balsa core to the B(±45) configuration and then to the B(±45/90) configuration leads to an increase in the compressive stiffness by approximately 400% and 600%, respectively (from 10 to 39 to 59 MPa), whereas the linear elastic compressive strength increases from about 560% to about 660% (from 1 to 5.6 to 6.6 MPa).

**Figure 6:**
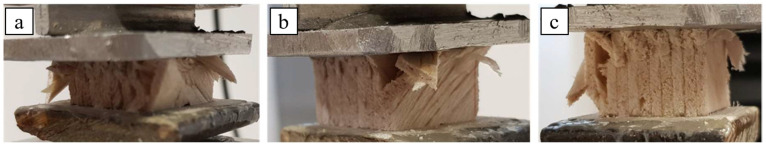
Typical fractures of core specimens tested in flatwise compression: (**a**) B(0), (**b**) B(±45), (**c**) B(±45/90).

In order to observe the peculiar damage mechanisms, Figure 6 shows the specimens during the flatwise compression test.

From Figure 6, it is possible to observe how, for all the tested specimens, significant phenomena of compressive buckling and subsequent delamination, intralaminar for B(0) and interlaminar for the other 2 cores, occur.

### 3.3. Structural Characterization of the Sandwich

The characterization of the sandwiches considered in this study was obtained by means of a rail shear test (RST) and a three-point bending test (TPB), in accordance with the relevant standards.

#### 3.3.1. Rail Shear Test

**Figure 7:**
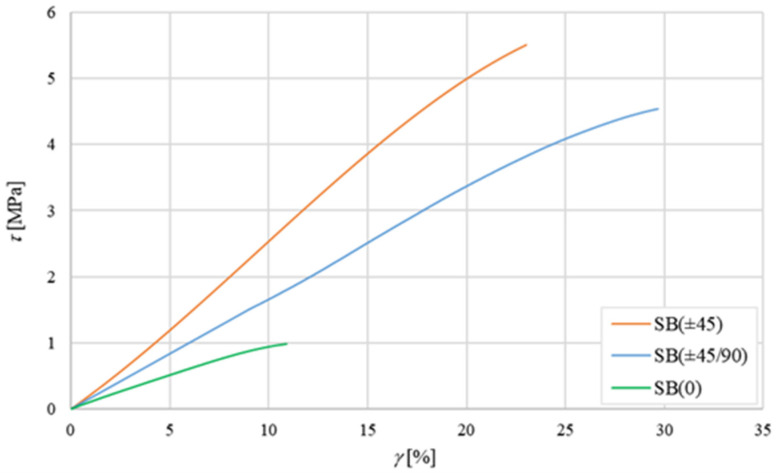
Average shear curves for sandwiches SB(0), SB(±45), and SB(±45/90).

The shear test was performed according to ASTM standard C273-61 [28] by using an HBM W 10 TK inductive transducer to measure the relative displacement of the two opposite faces of the sandwich. Using this test, the shear strength, the percentage creep at failure, and the elastic shear modulus can be obtained. The tests have been carried out under displacement control with a speed of 0.5 mm/min. For each type of sandwich, four separate specimens have been considered; Figure 7 shows the average experimental shear curves (*τ*–*γ*).

From Figure 7, a quasi-linear behavior with a shear modulus *G_c_* ≅ 10 MPa and a shear strength *τ_c_*_,*R*_ ≅ 1.0 MPa (failure shear strain *γ_c_*_,*R*_ ≅ 11%) is observed for the SB(0) sandwich. A similar curve with an almost linear behavior with shear modulus *G_c_* ≅ 25 MPa and a shear strength *τ_c_*_,*R*_ ≅ 5.5 MPa (failure shear strain *γ_c_*_,*R*_ ≅ 23%) is observed for the SB(±45) sandwich. Also, the SB(±45/90) sandwich exhibits almost linear behavior with a shear modulus of *G_c_* ≅ 15 MPa and shear strength *τ_c_*_,*R*_ ≅ 4.5 MPa (failure shear strain *γ_c_*_,*R*_ ≅ 29%). All these results are in good accordance with the micromechanics and the CTL [27] that predict the increment of the shear stiffness and the shear strength when the lamina orientation passes from 0 (or 90°) to ±45°.

As expected, the highest shear strength corresponds to the core with laminae arranged at ±45°, while the intermediate value of the core with laminae in the ±45/90 sequence, i.e., 4.5 MPa, is near to the expected value given by the weighted average of the laminae at 0° (1 MPa) and at ±45° (5.5 MPa). It is therefore confirmed that the use of laminar configurations instead of simple balsa panels allows the user an increase in shear performance of up to 550%.

**Figure 8:**
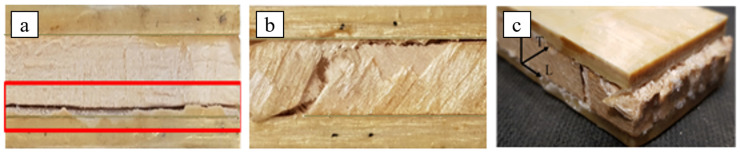
Shear failure of the sandwich core: (**a**) SB(0)_10_, (**b**) SB(±45)_10,_ and (**c**) SB(±45/90)_10_.

In order to confirm the shear failure modes, images of the three different sandwiches after the shear tests are shown in Figure 8 below:

In particular, from Figure 8, it is observed that the shear failure of the SB(0) sandwich occurs in the core, although in a zone near the interface (see red frame in Figure 8a). The shear failure of the SB(±45) sandwich, instead, involves ruptures at ±45° with coexistent interlaminar delamination phenomena that can lead to local delamination triggers at the skin-core interface (Figure 8b); also, the shear failure of the SB(±45/90) sandwich follows similar damage mechanisms (see Figure 8c).

The essential results of the shear tests are summarized in Table 2 below.

#### 3.3.2. Three-Point Bending Test

The flexural characterization tests of the sandwiches were performed in accordance with ASTM C393-00 [29]. Depending on the geometry and the applied load, from these tests it is possible to derive the flexural stiffness of the sandwich, the shear strength of the core, and the tensile/compression failure load of the skins.

Specifically, if *r* < *r_max_* (the details of the calculation of *r* and *r_max_* can be found in Appendix A), the core shear strength is given by the following formula [29]:

(1) $$\tau_{c,R}=\frac{P}{\left(h+h_{c}\right)b}$$

where *P* is the maximum load recorded during the test, *h* is the total thickness of the sandwich, *h_c_* is the thickness of the core, and *b* is the width of the examined sandwich specimen.

If instead *r* > *r_max_*, then the flexural ultimate stress of the skins is obtained by using the formula:

(2) $$\sigma_{s,u}=\frac{P\cdot L}{2t\left(h+h_{c}\right)b}$$

where *L* is the span length and *t* is the thickness of the skins.

In the present case, the specimens were subjected to a three-point bending test by using the same machine as in the previous test, suitably equipped with a pair of sliding supports for adjusting the desired span length (distance between the lower supports). In accordance with the standard, the test has been performed using a speed rate set at 1 mm/min; load and displacement data have been taken directly from the machine’s sensors.

Considering the typical thicknesses of synthetic sandwiches used in structural applications, generally in the range of 15–30 mm for each type of sandwich, two distinct core heights of 10 and 25 mm were considered to have sandwiches with a total thickness of 18 mm and 33 mm, being 4 mm the thickness of the skins. In order to also investigate the possible indentation phenomena, as well as possible corrugation and skin-core delamination, which are difficult to predict theoretically or numerically, specimens of length *L* = 200 mm were manufactured to always obtain the condition *L* < 2·*r_max_* (see Table 3), i.e., a loading condition characterized by high shear stresses that can easily trigger indentation, corrugation, and skin-core delamination phenomena. The dimensions of the specimens and their characteristics are summarized in Table 3 below:

##### Flexural Test of the SB(0)_25_ Sandwich

**Figure 9:**
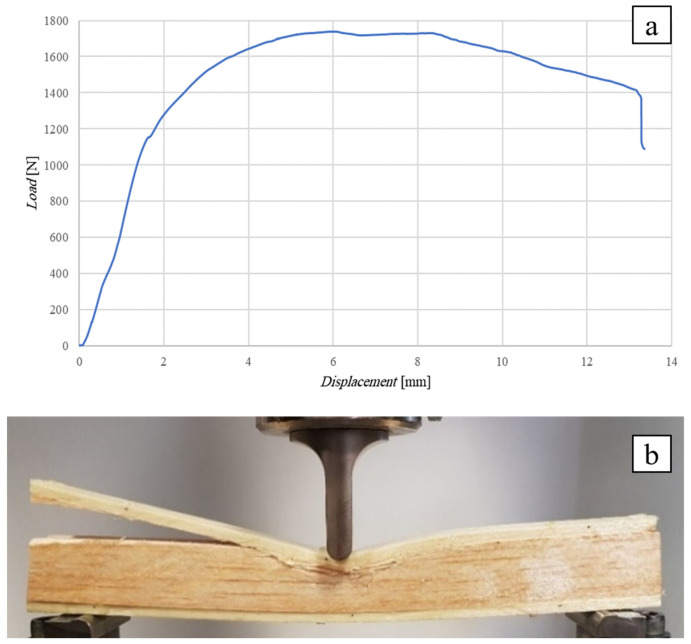
Flexural test of the SB(0)_25_ sandwich (**a**) flexural curve, (**b**) specimen at failure.

Figure 9 below shows the flexural curve (vertical displacement measured by the same testing machine) of the SB(0)_25_ sandwich, i.e., the sandwich with a 10 mm thick core of simple balsa wood, together with the image of the test specimen after failure. In order to highlight the effects of the possible indentation of the core around the point of application of the load, this was applied by using a cylindrical punch having a diameter of 10 mm.

Figure 9a shows how the sandwich exhibits an initial linear elastic behavior up to a load of approximately 1200 N, followed by an elasto-plastic behavior characterized by a progressive indentation of the core up to the core shear failure load of 1740 N. At this load level, the initiation in the middle section of a shear fracture of the core is experimentally observed, which proceeds towards the left support, first moving towards the interface (probably a relatively weak zone of the bond) and then returning to the core before reaching the end section (see Figure 9b).

The comparison of the actual failure load of 1740 N with the load *T_max_* = 1446 N theoretically evaluated on the basis of the core failure load *τ_c_*_,*R*_ = 1 MPa detected by the preliminary shear test shows an increase in about 20%; this result is probably due to the beneficial effects of the direct compression operated by the load on the shear failure mechanism of the core. The maximum stress on the skins, calculated using the inverse of Equation (A1), is *σ_s_*_,*max*_ = 35 MPa (see Appendix A), which is just about 35/310·100 = 11% of the skin ultimate stress, indicating a significant distance of the experimental failure conditions from the conditions of the sandwich failure due to the skin failure that would occur for four specimens with *L* > 2520 mm (see Table 3).

As a result of the experimental observation, it can be stated that in order to avoid local indentation of the core during service, it is necessary to recommend keeping the specific pressure due to the load below the value of 1200 N/(10 mm·21.5 mm) = 5.5 N/mm^2^, with a sufficient safety margin. As an example, if a service load of 10 kN has to be applied, then an application surface of 10.000/5.5 = 1.818 mm^2^ (a square of about 50 mm × 50 mm) has to be used.

##### Flexural Test of the SB(0)_10_ Sandwich

**Figure 10:**
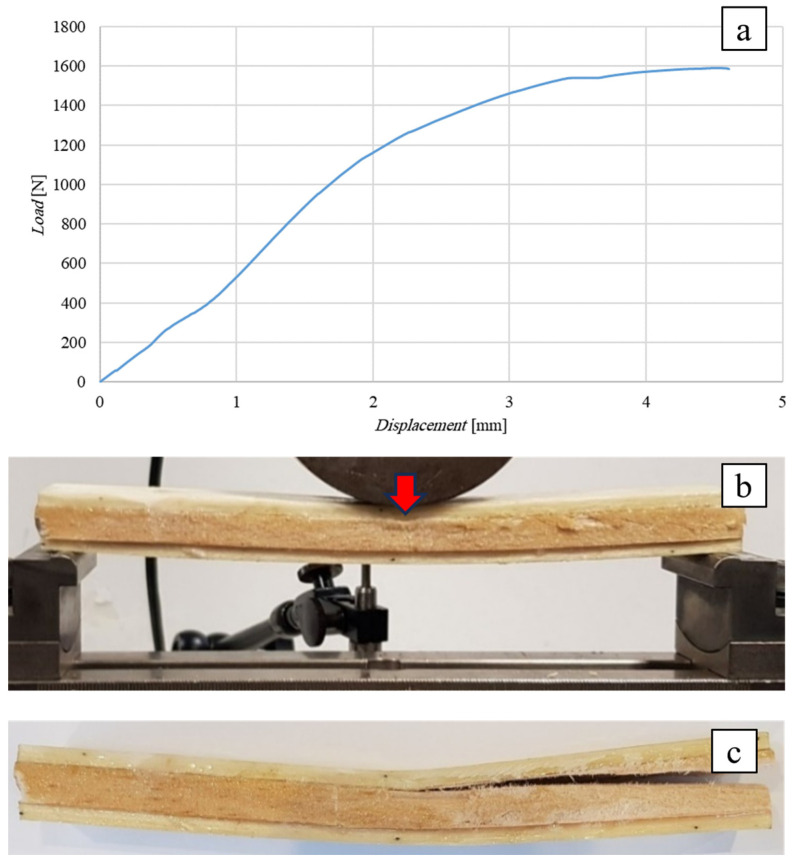
Flexural test of the SB(0)_10_ sandwich: (**a**) flexural curve, (**b**) specimen during, and (**c**) after the test.

The following Figure 10 shows the flexural curve (vertical displacement measured by means of a special LVDT-type displacement transducer) of the SB(0)_10_ sandwich, i.e., the sandwich with a 10 mm thick simple balsa core, together with the image of the specimen during the test (Figure 10b) and after failure (Figure 10c). In order to limit the indentation phenomena, a punch consisting of a large cylinder with a diameter of 80 mm was used (see Figure 10b).

Similar to the previous case, the test shows a linear elastic behavior up to a load of 1200 N, followed by an elasto-plastic behavior up to a failure load of approximately 1590 N corresponding to a displacement of 4.6 mm. The observation of the specimen during and after the test shows how the use of the large-diameter punch leads to negligible local indentation effects (see Figure 10b,c) and that the failure of the sandwich actually occurs by shear failure of the core, starting from the mid-section, near the upper skin-core interface, and propagating towards the point of intersection between the middle plane and the right end of the specimen.

Comparison of the experimental failure load value (1590 N) with the theoretical value of 1062 N (see Table 3) shows an increase in approximately 50%, which again can probably be justified by the positive effects of the direct compression caused by the applied load on the shear failure. The greater increase observed with respect to the SB(0)_25_ sandwich examined above is probably justified by the absence of indentation phenomena whose presence, as is easy to understand, gives rise to premature shear failure of the core.

Also in this case, the stress on the skins obtained from Equation (2), equal to σ* = 48 MPa, is far from the skin ultimate stress (about 15%), showing how, once again, the experimental failure condition is far from the condition corresponding to the failure of the sandwich due to the fracture of the skins, which would occur for specimen lengths *L* > 1960 mm (see Table 3).

##### Flexural Test of the SB(±45)_25_ Sandwich

**Figure 11:**
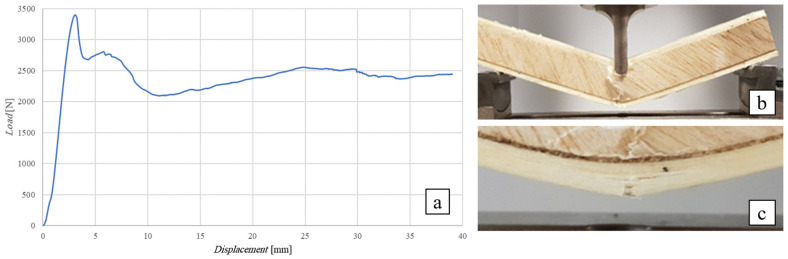
Flexural test of the SB(±45)_25_ sandwich: (**a**) flexural curve, (**b**) specimen during the test, and (**c**) after tensile failure of the lower skin.

The following Figure 11 shows the flexural curve (vertical displacement measured by the same material testing machine) of the SB(±45)_25_ sandwich, i.e., the sandwich with a 25 mm thick cross-ply laminate core, together with the image of the specimen during the test (Figure 11b) and after failure (see Figure 11c). As already performed for the B(0)_25_ sandwich, in order to highlight possible indentation phenomena and their consequences on the performance of the sandwich, the load was applied by using a cylindrical punch with a relatively small diameter of 10 mm.

Figure 11a shows how the sandwich exhibits linear elastic behavior up to failure, which occurs at a load of approximately 3400 N (displacement of about 3 mm), followed by a significant reduction in load of approximately 40% with an appreciable increase in deflection up to approximately 12 mm. This part of the curve corresponds in practice to the compression/shear failure of the skin (see Figure 11b) due to the conspicuous indentation phenomenon of the core, which stops at the occurrence of a sort of plastic hinge on the middle of the lower skin (the final pseudo-horizontal part of the flexural curve), resulting in the final failure of the sandwich (see Figure 11c). It can be seen that in this case, the high shear strength of the core makes it possible to reach a load that is about double the load reached by the sandwiches with simple balsa examined previously, although due to the high specific pressure equal to about 3400/(10·21.5) = 16 MPa, the consequent cutting of the upper skin and the deep indentation of the core give rise to a residual system in which the only resistant element is the lower skin, which yields by tensile stress, giving rise to the final failure of the sandwich.

This results in an early failure of the sandwich without the reaching of the theoretical failure load of 7795 N (see Table 3) related to the core shear failure, which does not occur experimentally at all since it is anticipated by the cutting of the skin, the indentation of the core, and the tensile failure of the lower skin.

Evidently, the local damage process observed indicates that for the SB(±45)_25_ sandwich, in order to avoid the cutting of the skin with subsequent severe indentation of the core, it is necessary to limit the specific pressure below the 16 MPa value estimated above. If this value is compared with that estimated for the SB(0)_25_ sandwich, it can be seen that the core laminar configuration at ±45 leads not only to a more than five-fold increase in shear strength (see comparison in Figure 7) but also to the tripling of the indentation strength, from 5.5 MPa to 16 MPa.

##### Flexural Test of the SB(±45)_10_ Sandwich

**Figure 12:**
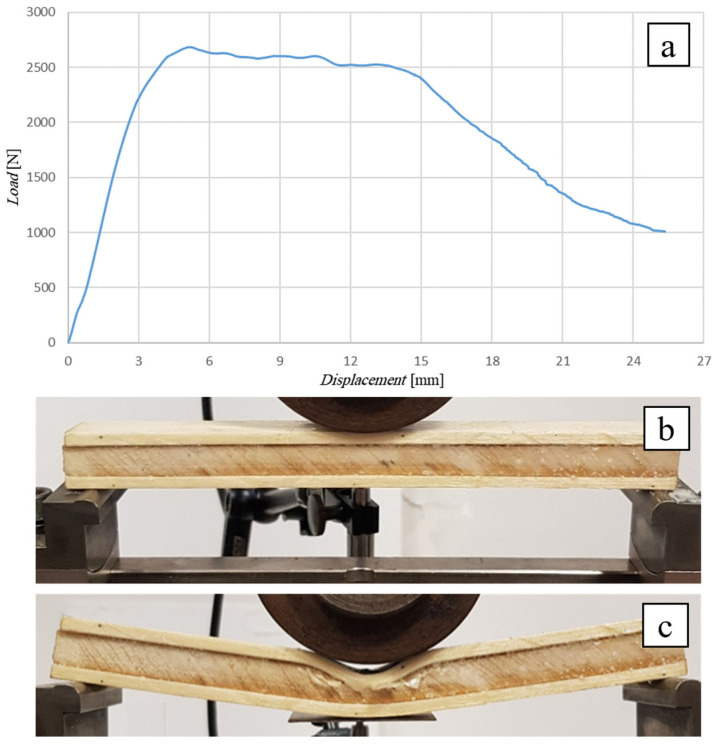
Flexural test of the SB(±45)_10_ sandwich: (**a**) flexural curve, (**b**) specimen during the test, and (**c**) after compression buckling of the upper skin.

The following Figure 12 shows the flexural curve (vertical displacement measured by means of an LVDT transducer) of the SB(±45)_10_ sandwich, i.e., the sandwich with a 10 mm thick cross-ply laminate core, together with the image of the specimen during the test (Figure 12b) and after failure (see Figure 12c). As already performed for the B(0)_10_ sandwich, in order to avoid possible indentation phenomena and their consequences on the performance of the sandwich, the load was applied by using a cylindrical punch with a diameter of 80 mm. It should be emphasized that for a fixed span length, the adoption of a lower core thickness makes it possible to increase the flexural stress on the skins, permitting the highlighting of the possible phenomena of sandwich failure due to corrugation.

From Figure 12a, it is possible to observe that the sandwich exhibits an almost linear elastic behavior up to the failure, which occurs in practice at a load of 2680 N, which is significantly lower (approximately −45%) than the theoretical load expected for core shear failure, equal to 4950 N (see Table 3). The initial linear phase is followed by a plateau (deformation under an almost constant load) associated in practice with an evident phenomenon of instability of the elastic equilibrium of the compressed upper skin, as it can be observed in Figure 12c. As expected, this phenomenon occurs at almost constant load until a final failure of the sandwich by tensile failure of the lower skin is triggered. The failure load of 2680 N corresponds to a compressive stress of the upper skin of approximately 97 MPa (Equation (2)), a value that can therefore be considered characteristic for the occurrence of possible buckling phenomena of the skins. This phenomenon is evidently responsible for the premature compressive failure of the core, which does not follow at all the expected shear failure. In other words, it is observed that although the risk of indentation of the core is eliminated by reducing the specific pressure below the limit value of 16 MPa detected in the previous test (thanks to the use of a large diameter punch), it is still not possible to reach the expected shear failure load of the core, since this expected failure mechanism is preceded by the instability of the elastic equilibrium of the compressed skin, which occurs for a relatively modest compressive stress of about 97 MPa, equal to about 1/3 of the 310 MPa failure load of the biocomposite. Certainly, the replacement of the natural balsa B(0) core with the innovative laminar B(±45) core permits an increase in the failure load of about 70% and 100% for sandwiches with a core of 10 mm and 25 mm, respectively; but in none of the cases is it possible to exploit the full advantage of the improved properties of the (±45) core since the shear failure of the core is preceded by the cutting of the upper skin followed by deep indentation of the core for SB(±45)_25_ sandwiches or by buckling of the compressed upper skin for SB(±45)_25_ sandwiches. In both cases, it is possible to state that the premature failure is linked to the low transversal stiffness of the core, which is certainly not sufficient to limit the indentation of the core and the instability of the upper compressed skin. This indicates as a possible solution the adoption of a laminar configuration presenting greater transversal compression strength, which can evidently be obtained by introducing in the ±45° laminate a 90° lamina at least, i.e., by adopting the proposed configuration [(±45/90)] considered below.

##### Flexural Test of the SB(±45/90)_25_ Sandwich

**Figure 13:**
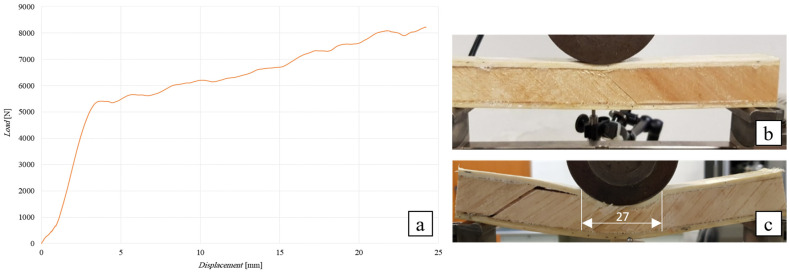
Sandwich flexural test SB(±45/90)_25_: (**a**) flexural curve, (**b**) specimen during the test, and (**c**) after shear failure of the core.

The following Figure 13 shows the flexural curve (vertical displacement measured by LVDT transducer) of the SB(±45/90)_25_ sandwich, i.e., the 25 mm thick angle-ply laminate core sandwich with lay-up [(±45/90)*_n_*], together with the image of the specimen during the test (Figure 13b) and after failure (see Figure 13c). To ward off possible indentation phenomena and their consequences on the performance of the sandwich, the load has been applied by using the cylindrical punch with a diameter of 80 mm.

From Figure 13a it is possible to observe a first linear behavior up to a load of about 5300 N, which is followed by a second part, which is also almost linear, corresponding to relatively limited phenomena of indentation of the upper skin (see Figure 13b) and then of the core, up to the failure load of about 8230 N, which corresponds in practice to complex damage constituted by a shear failure of the core (see Figure 13c), which occurs with intralaminar fractures parallel to the fibers and concomitant phenomena of interlaminar delamination and partial skin-core delamination. The comparison of the actual failure load of 8230 N with the expected shear failure load of the core, equal to 8477 N (see Table 3), shows a slight overestimation of 3%, which falls within the common scattering of similar experimental data; consequently, it is possible to state that the limited phenomena of pure indentation do not limit the mechanical performance of the examined sandwich. Taking into account the peculiar linearity of the flexural curve even after the indentation phenomena have been triggered, it is possible to state that during the ordinary use of the sandwich, these premature damage phenomena can be avoided by simply reducing the contact pressure below the value, which can be easily assessed from Figure 13c, which represents the specimen at the end of the first linear elastic phase, i.e., 5300/(17.5·28) = 11 MPa, with an adequate safety margin. Obviously, such an estimated value is higher than the flatwise compression strength of the core, equal to 6.5 MPa (see Figure 5), due to the beneficial effects of the greater transversal stiffness of the skins.

##### Flexural Test of the SB(±45/90)_10_ Sandwich

**Figure 14:**
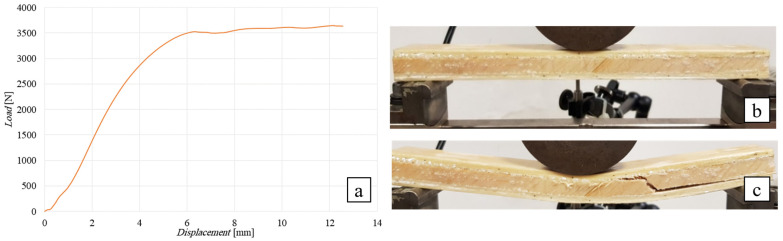
Flexural test of the SB(±45/90)_10_ sandwich: (**a**) flexural curve, (**b**) specimen during the test, and (**c**) after shear failure of the core.

The following Figure 14 shows the flexural curve (vertical displacement measured by LVDT transducer) of the SB(±45/90)_10_ sandwich, i.e., the 10 mm thick angle-ply laminate core sandwich with lay-up [(±45/90)*_n_*], together with the image of the specimen during the test (Figure 14b) and after failure (see Figure 14c). As above, the load has been applied by using a cylindrical punch having a diameter of 80 mm.

From Figure 14a, it can be observed how this sandwich exhibits a linear behavior up to a failure load of approximately 3650 N, which is followed in practice by a plateau that corresponds to the propagation of the damage towards the ends of the specimen (see Figure 14b). Shear failure of the core (see Figure 14c) occurs in practice in the same manner as described for the sandwich with a core height of 25 mm. The comparison of the actual failure load of 3650 N with the expected load of 3792 N for core shear failure (see Table 3) still shows a negligible reduction in approximately 4%, so that it can still be stated that the indentation phenomena observed are negligible.

In summary, it is possible to briefly observe that the proposed laminar core configuration with lay-up [(±45/90)*_n_*] corresponds in practice to the optimal configuration for the development of a high-performance green sandwich made up of high-performance biocomposite skins consisting of a green epoxy matrix reinforced with optimized sisalane agave fibers. Its use, in fact, makes it possible to optimize the performance of the sandwich by avoiding the occurrence of premature indentation damage and/or instability of the compressed skin, which can significantly limit the performance of such green sandwiches, as widely shown by the previous experimental analysis.

## 4. Discussion and Comparisons

**Figure 15:**
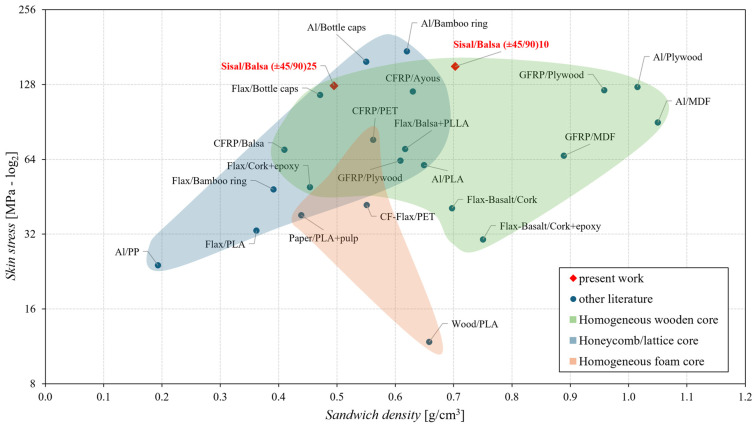
Comparison of the skin stress of the proposed green sandwich with that of other sandwiches reported in the literature [5,8,30,31,32,33,34,35,36,37,38,39,40,41,42,43,44].

In order to compare the mechanical performance of the innovative green sandwich proposed with that of other sandwiches available in the literature, by following the approach proposed in the review work [3], the graph of the skin stress vs. the structural density of the sandwich has been reported in Figure 15.

From such a figure, it can be observed how the proposed sandwich, having the optimal core configuration [±45/90], exhibits the best behavior in terms of skin stress if compared to other sandwich structures with cores of bio-based origin, mainly wood-based (green area) [5,8,30,31,32,33,34]. Also, the performance of the proposed sandwiches is comparable with that of the best sandwich structures with lightweight hybrid sandwich panels developed by replacing solid cores with honeycomb/lattice cores with bio-based polymer matrices or cores for fully recyclable panels (blue area) [35,36,37,38,39,40,41]; the proposed sandwich shows instead significantly better performance than sandwich structures made with foam cores (brown beige area) [42,43,44].

**Figure 16:**
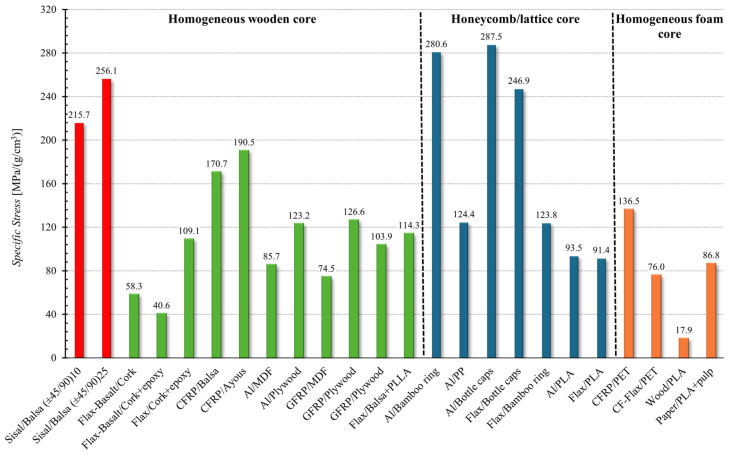
Comparison of the specific stress of the optimal green sandwich with that of other sandwiches reported in the literature [5,8,30,31,32,33,34,35,36,37,38,39,40,41,42,43,44].

Taking into account that sandwich structures are essentially materials used in lightweight applications where the relevant characteristic parameter is the specific stress, obtained by dividing the absolute skin stress by the specific weight, the following Figure 16 compares the strength of the proposed sandwiches with that of other sandwich panels present in the literature in terms of the specific stress.

From Figure 16, it is possible to observe how the proposed sandwich panel, with an optimized core [±45/90], presents specific stress values (215–256 MPa/(g/cm^3^)) significantly higher than sandwich structures with similar cores (homogeneous wooden core that fall in the range 40–200 MPa/(g/cm^3^)) and sandwich panels with foam cores (17–240 MPa/(g/cm^3^)), whereas they are comparable with the best performing sandwiches made with honeycomb cores (bamboo ring core and bottle caps core that take values about 280 MPa/(g/cm^3^)).

**Figure 17:**
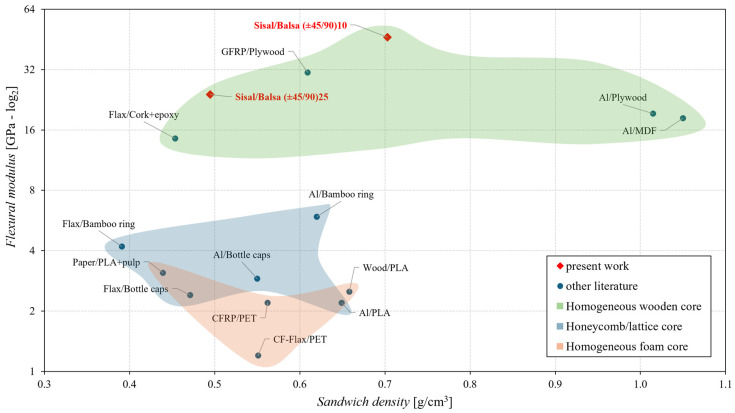
Comparison of the flexural modulus of the proposed green sandwich with that of other sandwiches reported in the literature [8,31,33,35,37,38,39,40,42,43,44].

**Figure 18:**
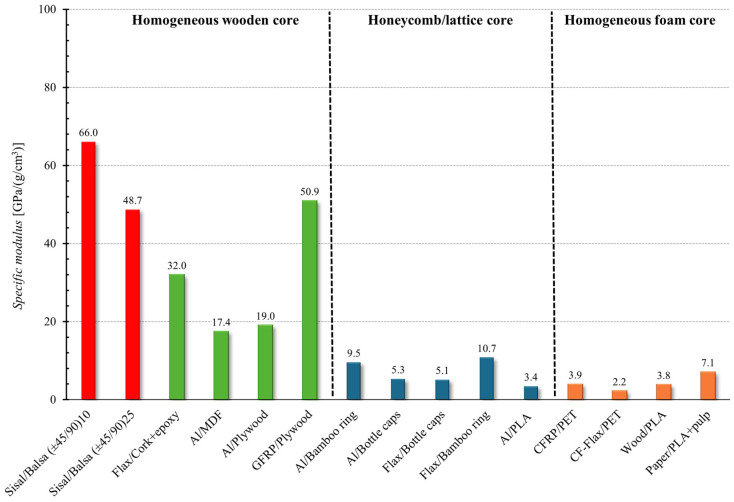
Comparison of the specific flexural modulus of the proposed green sandwich with that of other sandwiches reported in the literature [8,31,33,35,37,38,39,40,42,43,44].

Similarly, the following Figure 17 and Figure 18 show the comparison in terms of the absolute and specific flexural modulus, respectively.

From Figure 17, it is possible to note how the flexural modulus obtained for the proposed sandwich panel is at least one order of magnitude higher than the values present in literature for sandwiches with honeycomb/lattice (blue area) and foam cores (brown beige area), whereas it is comparable with the flexural modulus of the best-performing sandwiches having homogeneous wooded cores (green area).

In terms of specific modulus (see Figure 18), which is obtained by dividing the flexural modulus of the sandwich panel by its sandwich density, it is evident that the sandwiches proposed in this work are the most effective materials for lightweight structural applications. Additionally, thanks to the low specific weight of the constituent materials, as well as the low cost of sisal fibers, it can be stated that the green high-performance sandwiches implemented by the present work can be advantageously used for structural applications to replace synthetic sandwiches, with a significant reduction in the environmental impact as well as of weight and costs.

## 5. Conclusions

By means of a systematic experimental analysis, the present work has allowed the development of an innovative green sandwich for structural applications that can be advantageously used to replace synthetical sandwiches made of fiberglass skins and various cores. The proposed sandwich consists of high-performance biocomposite unidirectional skins, having a tensile strength of about 310 MPa, already extensively developed by the same authors in previous works by using a green epoxy matrix reinforced with optimized sisalana agave fibers and a renewable, innovative laminate balsa core. In particular, it has been proved that:

- the use of natural balsa gives rise to inefficient sandwiches, i.e., sandwiches characterized by low load-bearing capacities (included in the range 1590 ÷ 1740 N for sandwiches with core thicknesses of 10 ÷ 25 mm), mainly due to the limited shear strength of common balsa, equal to approximately 1 MPa;
- the mechanical efficiency improves instead significantly by using innovative cross-ply laminar cores with laminae oriented at ±45°; such a configuration in fact permit to increase the load-bearing capacities up to failure loads in the range 2680 ÷ 3440 N (+70% ÷ 100% respect to the simple balsa), although such actual performance are significantly limited respect to the theoretical ones computed by considering the actual core shear strength of 5.5 MPa, due to the unavoidable premature core indentation phenomena and the low transverse compressive strength responsible for significant core indentation effects and the cutting of the compressed skin, or its buckling for core thicknesses of 25 mm and 10 mm respectively;
- however, thanks to the improved transversal compressive strength, the optimization of the sandwich performance is fully obtained by using a core laminate lay-up [(±45/90)*_n_*]; indeed, experimentation shows how for such an optimal core configuration, no premature core indentation and/or buckling of the compressed skin no longer occur, and the sandwich strength (8230 N) reaches in practice the theoretical value (8477 N);
- also, such an optimal configuration permits us to quintuple the shear strength (from 1 MPa to about 4.5 MPa) and the transversal compressive strength (from 1 MPa to about 6.5 MPa) of the core, avoiding completely the premature damage phenomena that typically limit the performance of sandwich, such as the indentation of the core around the applied load (indentation that can also lead to the cutting of the compressed skin) and the corrugation/buckling of the upper skin subjected to axial compression, made also easier by the low transversal compressive strength of the common core materials;
- the comparisons with the sandwiches reported in the literature have shown that the skin stress of the proposed optimal sandwich is higher than both the homogeneous foam core and the homogeneous wooden core, and it is comparable with the best sandwiches having a honeycomb/lattice core. Similar results are obtained in terms of specific stress by considering the actual specific sandwich density;
- in terms of absolute and specific flexural modulus, instead, the performance of the proposed sandwich is significantly higher than that of the honeycomb/lattice core and foam core and comparable with the sandwich having a homogeneous wooden core.

Finally, thanks to the low specific weight of the constituent materials (approximately 145 kg/m^3^ for balsa and sisal fibers), as well as to the low cost of sisal fibers (<0.30 €/kg on the large market), and the excellent performance found for sandwiches with lay-up-optimized laminar cores [(±45/90)*_n_*], it can be stated that the green high-performance sandwiches implemented by the present work can be advantageously used for structural applications to replace synthetic sandwiches, with a significant reduction in the environmental impact, as well as of weight and costs.

## Figures and Tables

**Table 1:** Mechanical properties of the materials used.

Material	*ρ* [g/cm^3^]	*σ_R_* [MPa]	*τ_R_* [MPa]	*E* [GPa]	*ε_R_* [%]
Sisal fiber	1.45	685	19.8	40	1.75
Balsa wood	0.15	4.9	1.5	0.23	-
Green Epoxy	1.05	50	25	2.5	1.20

**Table 2:** Results of shear tests on the sandwiches considered.

Sandwich	*τ_c_*_,*R*_ [MPa]	*G_c_* [MPa]	*γ_c_*_,*R*_ [%]
SB(0)	1	10	11
SB(±45)	5.5	25	23
SB(±45/90)	4.5	15	45

**Table 3:** Data on skins, cores, and different sandwiches analyzed.

		Skins		Core		Sandwich		
	*b* [mm]	*E_s_* [MPa]	*σ_s_* [MPa]	*G_c_* [MPa]	*τ_c_* [MPa]	*M_max_* [Nmm]	*T_max_* [N]	2·*r_max_* [mm]
SB(0)_10_	26	22,000	310	10	1	521.119	1062	1960
SB(±45)_10_	22	22,000	310	25	5.5	440.947	4950	356
SB(±45°/90)_10_	21.5	22,000	310	15	4.5	517.800	3792	420
SB(0)_25_	21.5	22,000	310	10	1	911.646	1446	2520
SB(±45)_25_	21.5	22,000	310	25	5.5	911.646	7995	458
SB(±45°/90)_25_	28	22,000	310	15	4.5	1187.260	8477	560

**Figure A1:**
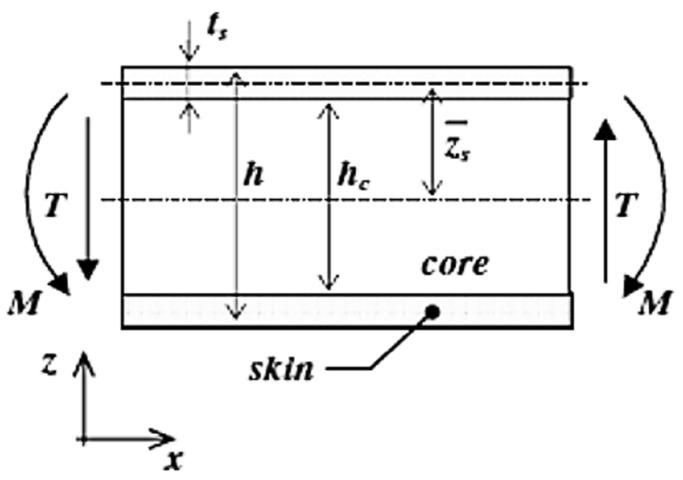
Sandwich panel sketch with geometrical and load notations.

## Data Availability

Data available on request from the corresponding author (due to privacy reasons).

## Appendix A. Failure Mechanisms and Strength Characterization

In order to proceed to a systematic development and a subsequent accurate mechanical characterization of a sandwich, it is necessary to take into account that, as it is well-known from the classical specialized literature [45,46,47] that consider sandwiches with synthetical composite skins (reinforced by glass, aramid or carbon fibers), the mechanical damaging of such structures can generally occurs through various mechanisms, as:

(1) skin compressive/tensile failure [48];
(2) core shear failure [49,50];
(3) delamination, i.e., skin–core debonding [51];
(4) local wrinkling of skins due to compression buckling [52,53];
(5) core indentation failure due to high specific pressure [54,55].

In general, for a failure of type (1) or (2), it is possible to make an analytical prediction [56,57] of the strength of the sandwich from the knowledge of the mechanical properties of skins and core. More specifically, by considering a generic loading condition consisting of a flexural moment *M* and the associated shear force *T* (see Figure A1), failure of the sandwich follows the failure of the skins that occurs when the maximum tensile stress $\sigma_{x,max}$ or compressive stress $\sigma_{x,max}^{’}$ acting on the outer surfaces of the skins exceeds the corresponding ultimate tensile stress $\sigma_{s,u}$ or ultimate compressive stress $\sigma_{s,u}^{’}$. Alternatively, the sandwich failure follows the shear failure of the core that occurs when the maximum shear stress $\tau_{xz,max}$, normally acting on the middle plane of the core, exceeds the corresponding ultimate shear stress $\tau_{c,u}$.

Considering the such two primary failure mechanisms of skins and cores and using the notation given in Figure A1, according to the Classical Theory of Laminates (CTL), the tensile/compressive failure of skins and the shear failure of the cores occurs when *M* and *T* exceed the corresponding *M_max_* and *T_max_* values given by:

(A1) $$M_{max}=\frac{2D}{E_{s}\;h}\sigma_{s,u}^{*}\;(\mathrm{skins}\;\mathrm{ultimate}\;\mathrm{flexural})$$

(A2) $$T_{max}=\frac{N}{G_{c}\;h}\tau_{c,u}\;(\mathrm{core}\;\mathrm{ultimate}\;\mathrm{shear}\;\mathrm{load})$$

where *E_s_* is the Young’s modulus of the skins, $\sigma_{s,u}^{*}\;$ is the minimum value between the tensile strength $\sigma_{s,u}$ and the compressive strength $\sigma_{s,u}^{’}$ of the skins, *G_c_* is the shear modulus of the core, *D* and *N* are the flexural and shear stiffnesses of the sandwich, respectively, given by the following formulae derived from CTL: (core ultimate shear load)

(A3) $$D=\frac{E_{s}\left(h^{3}-h_{c}^{3}\right)}{12\left(1-\nu_{s}^{2}\right)}$$

(A4) $$N=\frac{{G_{c}\left(h+h_{c}\right)}^{2}}{4h_{c}}$$

It should be noted that, in general, except for the phenomena of elastic equilibrium instability, the compressive strength of laminated composite skins is generally greater than the tensile strength; consequently, in the presence of pure flexural, the failure of the skin subjected to tensile load generally anticipates the failure of the opposite skin subjected to compressive load. Consequently, from Equations (A1) and (A2) it follows that in the presence of a generic flexural and shear load, the failure of the composite sandwich is caused by the failure of the skin subjected to tensile load, when the load ratio *r* = *M*/*T* is greater than the corresponding critical value *r_max_ = M_max_*/*T_max_*, which considers the simultaneous failure of the skins (tensile failure) and core (shear failure), i.e.,

(A5) $$r=\frac{M}{T}>r_{max}=\frac{G_{c}}{E_{s}}\frac{2D}{Nh}\frac{\sigma_{s,u}}{\tau_{c,u}}$$

Otherwise, if the ratio *r* is less than *r_max_*, then sandwich failure is due to shear failure of the core, unless premature failure mechanisms such as indentation due to high specific pressure produced by the concentrated load, corrugation of the compressed skin due to instability under compression, or skin-core delamination due to poor performance of the adhesive or poor adhesion between adhesive and core materials and skins, are involved.

In the presence of common loads orthogonal to the mid-plane of the sandwich, the ratio r depends in practice on the geometry of the structure and on the constraint conditions. For example, if the sandwich is subjected to three-point bending (TPB), then the characteristic ratio *r* corresponds to half the so-called span length *a*_1_ [43], and failure is due to skin failure if the span *a*_1_ of the beam satisfies the condition:

(A6) $$a_{1}>2r_{max}\;\left(TPB\right)$$

Otherwise if Equation (A6) not satisfied, the sandwich is in practice a short beam and its failure is always due to the core failure. Unfortunately, due to the possible other damage mechanisms, that are difficult to predict theoretically (corrugation, indentation, delamination, etc.), the actual performance of a sandwich can be also very lower than that predicted by Equations (A1) and (A2). Consequently, a reliable assessment of the mechanical performance of a sandwich generally requires a systematic experimental analysis, the results of which can be used to calibrate theoretical strength prediction methods or even reliable numerical approaches [58,59].
